# The Use of Local and Global Ordering Strategies in Number Line Estimation in Early Childhood

**DOI:** 10.3389/fpsyg.2018.01562

**Published:** 2018-09-18

**Authors:** Jaccoline E. Van ’t Noordende, M. J. M. Volman, Paul P. M. Leseman, Korbinian Moeller, Tanja Dackermann, Evelyn H. Kroesbergen

**Affiliations:** ^1^Department of Child Development and Education, University of Amsterdam, Amsterdam, Netherlands; ^2^Department of Special Education: Cognitive and Motor Disabilities, Utrecht University, Utrecht, Netherlands; ^3^Leibniz-Institut für Wissensmedien, Tübingen, Germany; ^4^Department of Psychology, Universität Tübingen, Tübingen, Germany; ^5^Behavioural Science Institute, Radboud University Nijmegen, Nijmegen, Netherlands

**Keywords:** numerical development, number line estimation, strategy use, local ordering, global ordering

## Abstract

A lot of research has been devoted to number line estimation in primary school. However, less is known about the early onset of number line estimation before children enter formal education. We propose that ordering strategies are building blocks of number line estimation in early childhood. In a longitudinal study, children completed a non-symbolic number line estimation task at age 3.5 and 5 years. Two ordering strategies were identified based on the children’s estimation patterns: local and global ordering. Local ordering refers to the correct ordering of successive quantities, whereas global ordering refers to the correct ordering of all quantities across the number line. Results indicated a developmental trend for both strategies. The percentage of children applying local and global ordering strategies increased steeply from 3.5 to 5 years of age. Moreover, children used more advanced local and global ordering strategies at 5 years of age. Importantly, level of strategy use was related to more traditional number line estimation outcome measures, such as estimation accuracy and regression fit scores. These results provide evidence that children use dynamic ordering strategies when solving the number line estimation task in early stages of numerical development.

## Introduction

The oldest known illustration of a number line was published in 1685 in John Wallis’ book “Treatise of Algebra.” The concept of the number line was an unconventional idea in the 17th century (Núñez, 2011). Its use increased over time and number lines are nowadays commonly used in research and practice. With its increased use, there has also been an increase in theoretical models and analysis methods to evaluate performance on number line tasks. Most of these models were tested in primary school children and adults. However, numerical skills develop even before formal schooling starts (see Raghubar and Barnes, 2017, for a review on the development of early numerical skills). Nevertheless, our knowledge about the development of number line estimation at preschool age is still rather patchy. However, understanding the processes in the early stages is necessary to identify building blocks of later number line estimation performance. Children below 5 years of age are usually not able to estimate the position of symbolic Arabic numbers on a number line, because they do not yet know these numbers, but they may be able to estimate the position of numerosities in a so-called non-symbolic number line estimation task (cf. Kolkman et al., 2013). In the current study, 3.5- to 5-year-old children’s performance in non-symbolic number line estimation was evaluated with the aim of identifying building blocks of later number line estimation performance. We propose a new method to evaluate children’s estimation patterns based on ordering strategies.

### The Number Line Estimation Task

In number line estimation tasks, children usually have to estimate the spatial position of numbers on an otherwise empty number line. This number line is usually marked with a numerical start- and end-point (e.g., 0 and 100), although there are also unbounded versions of the number line task (e.g., Cohen and Blanc-Goldhammer, 2011; Cohen and Sarnecka, 2014; Link et al., 2014; Reinert et al., 2015; Opfer et al., 2016). Traditionally, symbolic numbers (i.e., Arabic numerals) are used in number line estimation tasks, but recently non-symbolic quantities (e.g., sets of dots) were used as well (e.g., Kolkman et al., 2013; Fazio et al., 2014; Friso-van den Bos et al., 2014b; Sasanguie et al., 2016). The current study focuses on bounded non-symbolic number line estimation.

Using non-symbolic quantities provides the opportunity to use number line estimation tasks in young children who do not yet master symbolic (Arabic) numbers. Nevertheless, the development of non-symbolic number line estimation has only been studied in children from age 5 years onward (Sasanguie et al., 2012a,b, 2013; Praet and Desoete, 2014; Sella et al., 2015). Most of these studies are based on research on symbolic number line estimation as regards to theoretical background, but also analysis methods are generalized from symbolic to non-symbolic number line estimation. One of the outcome measures used for both symbolic and non-symbolic number line estimation tasks is estimation accuracy, typically operationalized by the percentage absolute error of estimation. This score represents the deviation between participants’ estimates and the spatially correct position of the target numbers on the number line. Estimation accuracy was found to increase with age on both symbolic and non-symbolic number line tasks (Siegler and Booth, 2004; Berteletti et al., 2010; Reeve et al., 2015). However, there is an ongoing debate on the underlying cognitive mechanisms that lead to this increase in estimation accuracy. There are two main theoretical accounts: the “mental number line” and the “proportional reasoning” account.

### Theoretical Accounts of Number Line Estimation

The mental number line account states that number line estimation performance reflects the underlying mental representations of number magnitude (Siegler and Booth, 2004). This account is based on suggestions by Dehaene (1997, 2001), who states that the basis of numerical cognition is an innate representation of number magnitude in the form of a mental number line. This mental number line was suggested to be logarithmically compressed in those without experience with numbers or education (e.g., Pica et al., 2004), resulting in a characteristic estimation pattern: lower numbers are placed farther away from each other than larger numbers and thus, placement of the numbers on the number line becomes more dense with increasing numbers. According to this view, such a representation of number magnitude is reflected in a logarithmic distribution of young children’s estimates in number line estimation (Siegler and Booth, 2004). Such a logarithmic estimation pattern has been found in both symbolic and non-symbolic number line estimation tasks in 5- to 7-year-old children (Praet and Desoete, 2014). Through experience and education children learn that numbers are equidistant, which means that the distance between two adjacent numbers is always the same (e.g., the distance between 1 and 2 is equal to the distance between 91 and 92). Accordingly, this results in a linear distribution of children’s estimates along the number line (e.g., Siegler and Booth, 2004). The time point of the shift from a logarithmic to a linear estimation pattern was observed to depend on the number line format and the number range assessed. Praet and Desoete (2014) showed that second graders’ estimation patterns on a number line estimation task using (symbolic) Arabic digits fitted best to a linear model, whereas estimation patterns of the same children on a number line estimation task using (non-symbolic) dot patterns fitted best to a logarithmic model. This suggests that the shift from a logarithmic to a linear estimation pattern will take place earlier for (symbolic) Arabic numbers than for (non-symbolic) dot patterns. Other studies showed that the shift also takes place earlier for smaller than for larger number ranges. For example, in the study of Siegler and Opfer (2003), the best fitting model on second and fourth graders’ estimates was linear on a 0–100 number line task but logarithmic on a 0–1000 number line task. Therefore, Siegler and Opfer (2003) proposed that multiple mental number representations may coexist at the same time.

The existence of multiple estimation patterns within an age group was confirmed by the study of Bouwmeester and Verkoeijen (2012). However, they did not find a developmental trend from logarithmic to linear estimation patterns from the age of 5–8 years. Some younger children showed quite accurate (linear) estimation patterns on a symbolic 0–100 number line task, whereas some older children showed inaccurate estimation patterns. Moreover, although Bouwmeester and Verkoeijen (2012) did find a group of children showing estimation patterns resembling a logarithmic distribution, estimation patterns fitted better to a cubic model than to a logarithmic model. Estimation patterns of this group of children showed accurate estimates for numbers close to the beginning, midpoint, and endpoint of the number line, which suggests use of proportional reasoning.

The proportional reasoning account argues that participants’ number line estimation performance is not a direct reflection of their mental representation of number magnitude. Instead, it claims that number line estimation performance is influenced by proportional reasoning strategies used to solve the task (Cohen and Blanc-Goldhammer, 2011; Bouwmeester and Verkoeijen, 2012; Cicchini et al., 2014; Huber et al., 2014; Hurst et al., 2014). This account implies that participants use reference points (e.g., the middle of the line reflecting the position of 50 for a number line ranging from 0 to 100) to guide their estimates, which has been tested by applying power models to number line estimation data (Barth and Paladino, 2011; Cohen and Blanc-Goldhammer, 2011; Slusser et al., 2013; Rouder and Geary, 2014). For example, Rouder and Geary (2014) found that first graders’ estimates on a 0–100 number line task were fitted best by a one-cyclic power model reflecting the use of the beginning and endpoint of the line as reference points for estimation. Second graders’ consideration of the midpoint of the line as an additional reference point was reflected by a two-cycle power model (Rouder and Geary, 2014). Contrary to the mental number line account, the proportional reasoning account thus assumes that the estimates are actually formed *during* the task, and can even be influenced by specific task characteristics like the presence of external benchmarks on the number line (Peeters et al., 2017a,b).

The mental number line and the proportional reasoning account were tested against each other in research on symbolic number line estimation. Cyclic power models usually provided a better fit to number line estimation patterns than linear and logarithmic regression models from first or second grade and onward (Barth and Paladino, 2011; Slusser et al., 2013; Rouder and Geary, 2014; Friso-van den Bos et al., 2015). Logarithmic and linear estimation patterns seem to be caused by task characteristics (like the use of a bounded number line) instead of underlying mental representations, and the proportional reasoning account could provide an alternative explanation to seemingly logarithmic and linear estimation patterns (Cohen and Sarnecka, 2014; Cohen and Quinlan, 2018). For example, the developmental shift from logarithmic to linear estimation patterns could be explained by development in using proportional reasoning strategies (e.g., from the use of only the beginning and endpoint of the line as reference points, toward additional use of the midpoint of the line as a reference point), instead of development in underlying mental representations (Cohen and Sarnecka, 2014). Nevertheless, Dackermann et al. (2015) argued that neither one account nor the other may be sufficient in itself to fully explain children’s performance in number line estimation. Instead, they propose that number line estimation performance builds on both number magnitude representations and proportional reasoning. Moreover, they argue that familiarity with and understanding the characteristics of numbers is also essential to number line estimation. Several studies showed that numerical familiarity and understanding can even be a valid alternative explanation of seemingly logarithmic estimation patterns (e.g., Ebersbach et al., 2008; Stapel et al., 2015). For example, Ebersbach et al. (2008) demonstrated a link between children’s counting range (i.e., the range of numbers children could count correctly) and their estimates in number line estimation. Children were able to estimate numbers correctly on the number line as long as the numbers fell within their counting range. It seems reasonable to assume that it is not the mere knowing of the number words and their sequence that enhances number line estimation performance, but the understanding of the numerical magnitudes of the respective numbers. As such, it might be a combination of children’s understanding of ordinality and cardinality of numbers that is important to number line estimation. In this context, ordinality refers to understanding the position of numbers in relation to other numbers, whereas cardinality refers to understanding the actual magnitude of numbers. As indicated above, understanding both ordinality and cardinality are supposed to corroborate accurate estimations on a number line.

### Ordering Strategies in Number Line Estimation

The role of ordinality and cardinality in young children’s number line estimation performance was investigated by Sullivan and Barner (2014). They showed that kindergartners already understand the ordinal relation between numbers, even before they are able to make correct cardinal estimates on a symbolic number line ranging from 0 to 100. In particular, Sullivan and Barner (2014) examined whether children estimated each number in relation to the preceding number (i.e., the number that was presented directly before the current number). For example, a child first estimated the target number 30 to be located at the position of about 50 on the number line. Next, the target number 40 had to be estimated. In case the child already understands the ordinal relation between numbers 30 and 40, she/he should be able to estimate the location of 40 more rightward on the number line (i.e., somewhere between 50 and 100), even though this would not be the correct cardinal position relative to the beginning and endpoint of the number line. Sullivan and Barner (2014) found that 5-year-old children produced correct ordinal responses on about 70% of the trials, 6-year-olds on 84% of the trials and 7-year-olds on 93% of the trials, regardless whether they placed the target numbers at the correct cardinal position on the number line. Five-year-olds did make these correct ordinal responses without taking into account the correct relative distance between numbers (how far the number is positioned to the right or left of the preceding number), whereas 6- and 7-year-olds did consider relative distance between numbers. Moreover, many of the children did not only make correct ordinal responses in relation to the directly preceding number, but to almost *all* previously estimated numbers. This indicates that children do not only use trial-by-trial ordering, but also monitor their global ordering of numbers across the line on symbolic number line estimation.

A recent study by Cicchini et al. (2014) confirmed that trial-by-trial ordered responses were observed for non-symbolic number line estimation in 8- to 11-year-old children and adults as well. However, Cicchini et al. (2014) did not assess global ordering. Therefore, it is not yet clear whether both local and global ordering are used in non-symbolic number line estimation as well. Moreover, so far studies evaluating local and global ordering in symbolic and non-symbolic number line estimation only investigated children from 5 years of age and adults. The current study will be the first to examine whether children already use either/or both local (trial-by-trial) and global ordering strategies on non-symbolic number line estimation before they enter primary school.

### The Current Study

The aim of the current study was to evaluate the early onset and development of strategy use in number line estimation. Therefore, we evaluated estimation performance on a non-symbolic number line estimation task longitudinally in children from 3.5 to 5 years of age. In particular, we explored a new method of analyzing children’s estimation patterns, based on local and global ordering strategies. Local ordering refers to strategies considering response to preceding trials as reference points whereas global ordering refers to strategies reflecting an increasingly left-to-right ordering of increasing quantities across the number line (cf. Sullivan and Barner, 2014). Similar to the proceeding of Sullivan and Barner (2014), we only focused on (correct) ordering of quantities when coding strategy use, and not on correct cardinal positions on the number line.

In line with Sullivan and Barner (2014), we expected a developmental trend for local ordering strategies, from using only ordinal information (whether the target number should be placed to the right or left of the preceding target number) toward taking into account relative distance between quantities (how far to the right or left of the preceding target number). Additionally, we hypothesized a developmental trend for global ordering strategies as well. In the end, all participants should be able to correctly order all estimates across the number line (cf. Sullivan and Barner, 2014), but young children may not yet able to do this. Nevertheless, we hypothesized that young children should be able to use a basic level of global ordering, when ordering small quantities without differentiating between larger quantities (cf. Moeller and Nuerk, 2011).

To be able to correctly position quantities on the number line, both local and global ordering are probably needed. Therefore, we expected that the development in local and global ordering should be associated. Furthermore, increasing levels of both local and global ordering strategy use should lead to more accurate estimations of quantities on the number line. We used this hypothesis to test the validity of local and global ordering strategies as indications of number line estimation performance, by relating strategy use to more traditional measures of number line estimation such as absolute estimation error and regression fit scores.

## Materials and Methods

### Procedure

The current study was part of a larger longitudinal study^1^, consisting of two cohorts, followed from age 7 months to 3.5 years and from age 2.5 to 5 years, respectively. Data collected at age 3.5 and 5 years was considered in the current study. This enabled us to evaluate early onset and development of children’s strategies in number line estimation, just before children entered kindergarten at the age of 4 years, and follow this development into kindergarten.

Participants were recruited through the local government. The local government provided addresses of all parents with children in the eligible age range. An invitation letter was sent to all of these parents. Additionally, a small number of parents were recruited through Internet forums on parenting or via friends and family. For each cohort, 60 children with no indications of physical or mental health problems and born on-term (≥37 weeks of gestation) were selected to participate. Participants were selected based on order of application. Written informed consent was obtained from the parents of all children participating in the study. The study was approved by the local ethical research committee.

Data collection took place at our lab by trained master’s students following a fixed protocol. Parents were allowed to be present during the entire session, but they were instructed not to give any help to the child to complete the tasks.

### Participants

Forty-eight children from cohort 1 and 52 children from cohort 2 participated at age 3.5 years. Data from both cohorts were pooled for the current study, which resulted in a total sample of 100 children. Three children did not complete the number line estimation task and were therefore excluded from analyses. The remaining sample consisted of 63 girls (65%) and 34 boys (35%) at time 1. Their mean age was 3.60 years (SD = 0.06 years). Seventy-eight children (80%) were from higher educated families (higher vocational training or university completed).

Forty-five children from cohort 2 were tested again at age 5 years (mean age = 4.94 years, SD = 0.04 years). All children attended kindergarten at that time. Two of these children did not have data at 3.5 years (due to the child’s non-compliance and due to non-participation because of mother’s pregnancy) and were only included in the data analyses at 5 years. Thus, the follow-up sample consisted of 32 girls (71%) and 13 boys (29%). Thirty-nine (87%) children were from higher educated families.

### Instruments

An adapted version of the non-symbolic number line task of Kolkman et al. (2013) was used. A line of 1,000 pixels was presented on a computer screen run at a resolution of 1,280 by 1,024 pixels. Only the beginning and endpoint of the number line were marked, with 0 and 100 dots, respectively, throughout the entire task. These quantities were used as a way for children to make sense of the continuum, but were not introduced to the child as the specific numerical quantities “0” and “100.” Instead, the experimenter introduced the number line to participants as a road and target quantities as drops of gasoline needed for a car to drive along the road, using terms like “nothing,” “a little,” “a little more,” and “very much.”

First, the experimenter presented the child the quantity of 0 and told that the car could not drive without gasoline, and would therefore remain at the startpoint of the road. Next, the experimenter presented a small quantity to the child and pointed out that the car could drive along a small part of the road with this small amount of gasoline. A larger quantity was then presented and the experimenter pointed out that with a larger amount of gasoline the car could drive further along the road. And finally, a quantity of 100 dots was presented and the child was shown that with this large amount of gasoline the car could drive to the end of the road.

Following this instruction, we used four practice trials, in which children had to position quantities, including “0” and “100,” upon the number line, to make sure that they understood the concept of the number line.

**FIGURE 1:**
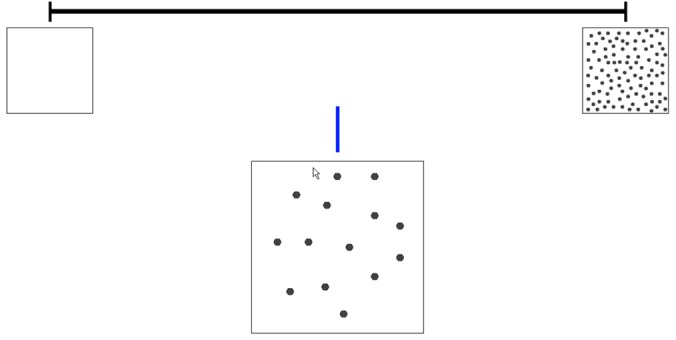
Example of a trial on the non-symbolic number line estimation task.

After practice, participants had to estimate the spatial position of 14 target quantities on the number line. These target quantities (6, 14, 21, 27, 33, 39, 47, 52, 59, 71, 76, 84, 90, and 95) were randomly selected, reflecting an equal distribution across the number range 0–100. The same quantities were used for all participants, but presented in random order. **Figure 1** shows an example of a trial. Quantities to be estimated were presented as dots inside a box below the number line. Dots were equal in size throughout the entire task. As young children might have problems using a computer mouse cursor, participants had to point out the spatial position of each quantity on the number line using his/her finger. The experimenter than dragged the mouse cursor to the position the child indicated.

### Analyses

#### Coding of Strategy Use

Individual estimation patterns were inspected to code the individual level of local and global ordering strategy use. For both strategies, levels were chosen to be mutually exclusive and higher levels were always preferred over lower levels.

##### Local ordering

To code local ordering strategy use, each estimate was related to the directly preceding estimate to examine whether the ordering of the quantities along the line was correct. Order was considered correct when the estimate was placed correctly to the right or left of the directly preceding estimate on the line. For example, when the first target quantity was 47 and the second target quantity was 33, the second estimate had to be located to the left of the first estimate, regardless whether both estimates were at the correct cardinal position on the line. Note that the target quantities were presented in random order and successive quantities thus differed between children. When the estimate was at about the same position as the previous estimate (i.e., within a 5% range of the number line around the previous estimate’s position), it was considered correct in case the numerical difference between the target quantity and the preceding quantity did not exceed 10 (10% of the number line’s numerical range). For example, positioning the target quantity 90 and the successive target quantity 95 at the same position was considered correct.

**FIGURE 2:**
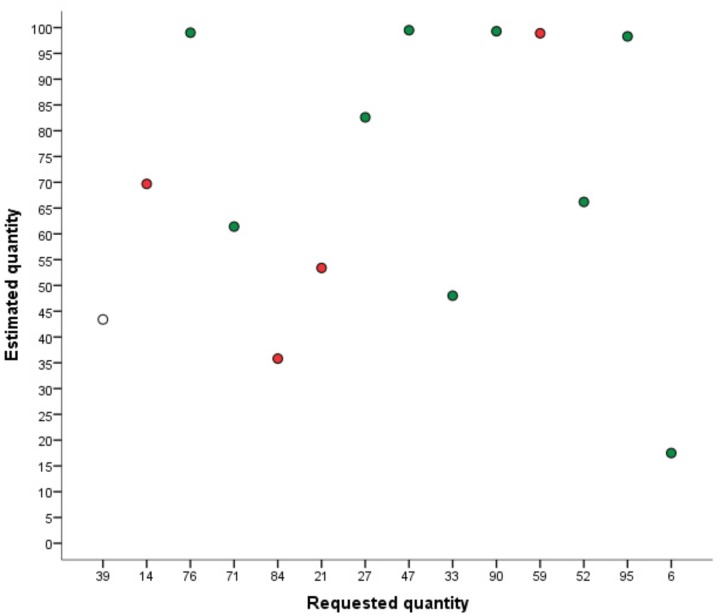
Example of local ordering. The *X*-axis shows the order of target quantities. Red dots represent incorrectly ordered estimates compared to the directly preceding estimate. Green dots represent correctly ordered estimates.

The following levels of local ordering were distinguished (see **Figure 2**):

0. No local ordering
  Less than half of the trials were in the correct order compared to the preceding estimate (<7 estimates).
1. Local ordering
  More than half of the trials were in the correct order compared to the preceding estimate (≥7 estimates).
2. Local ordering with relative distance of ±20%
  Similar to level 1, but in addition relative distance between correctly ordered successive estimates did not deviate from the correct relative distance between actual target quantity and preceding target quantity by more than ±20% of the number line’s numerical range. For example, when the first target quantity was 21 and the second target quantity was 52, the correct relative distance is 31. To fulfill the requirements of this level, the difference between the first and the second estimate has to be between 31 ± 20 = 11–51, regardless whether both estimates are at the correct cardinal position on the number line. In this case, for example, the first estimate could be 26 and the second estimate 46, resulting in a relative distance of 20, which falls in the range of a tolerated relative distance of 11–51.
3. Local ordering with relative distance 10%
  Similar to level 2, but the relative distance between correctly ordered successive estimates did not deviate from the correct relative distance between actual target quantity and preceding target quantity by more than ±10% of the number line’s numerical range. For example, when the numerical difference between the target quantity and the preceding target quantity was 20, the difference between the estimated quantity and the preceding estimated quantity had to be between 10 and 30.

##### Global ordering

**FIGURE 3:**
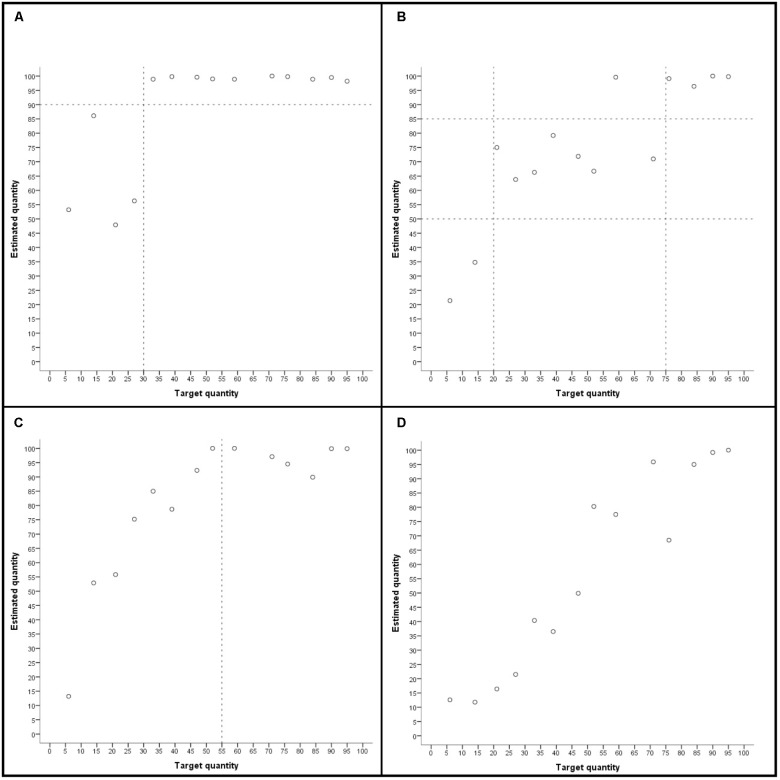
Examples of global ordering level 1 **(A)**, level 2 **(B)**, level 3 **(C)**, and level 4 **(D)**. Dashed lines indicate the distinction between small, medium, and large quantities.

In addition to local ordering, estimation patterns of each child were also inspected for the level of global ordering strategy use. A level was assigned when the majority of estimates met the description of the level given below. Four outliers (30%) that did not fit the estimation pattern were allowed, as long as a clear pattern meeting the level’s criteria was still visible. The following levels of global ordering were distinguished (see **Figure 3**):

0. No global ordering
  Estimates did not show a pattern of global numerical ordering; there was no correct distinction between lower and higher quantities (e.g., all estimates were at about the same position on the line).
1. Global ordering small/large
  Smaller quantities and larger quantities were distinguished and grouped together on the number line. The group of larger quantities was positioned to the right of the group of smaller quantities, but the cardinal position of the two groups of estimates was not considered. Identification of groups of smaller and larger quantities was based on visual inspection requiring that two groups of (small and larger) quantities could be clearly distinguished. Therefore, ranges and (cardinal) position of groups of quantities could differ between children.
2. Global ordering small/medium/large
  Similar to level 1, but quantities were grouped in three groups from left to right on the number line differentiating small, medium, and large quantities.
3. Global ordering small quantities
  Smaller quantities were ordered consecutively, whereas larger quantities were grouped together and not differentiated any further. Larger quantities were positioned to the right of smaller quantities, but cardinal position of estimates was not considered.
4. Global ordering all quantities
  The whole range of quantities was ordered consecutively, with larger quantities placed to the right of smaller quantities. Cardinal position of estimates was not considered.

### Statistical Analyses

#### Strategy Use and Development

After coding individual estimation patterns, the results were first analyzed for both time points separately. A frequency distribution indicated the number of children that used the respective strategy levels. Because we hypothesized that local and global ordering strategies *together* are building blocks of number line estimation, the interrelation between the two strategies was also evaluated, using Kendall’s tau-b correlation coefficient^2^.

Next, the development in strategy use from 3.5 to 5 years was investigated for both strategies separately. Wilcoxon signed rank tests were used to evaluate whether the level of local and global ordering strategy use was higher at 5 years than at 3.5 years of age. Kendall’s tau-b correlation coefficients were used to evaluate whether level of strategy use at 3.5 years correlated with level of strategy use at 5 years of age.

Finally, the interrelated development of local and global ordering strategies was evaluated. Kendall’s tau-b correlation coefficient was used to evaluate the association of the development in local ordering strategies from age 3.5 to 5 years with the development in global ordering strategies. Development in strategy use was indicated by a difference score subtracting the level of strategy use at 3.5 years from the level of strategy use at 5 years of age. Next, for each time point, each possible combination of the two strategies was assigned to one of seven groups with increasing competence level, by adding the level of local ordering strategy use to the level of global ordering strategy use (e.g., when a child used local ordering level 1 and global ordering level 2, her/his level of strategy combination would be 3). Kendall’s tau-b correlation coefficients were used to evaluate the relation between the combined level of strategy use at 3.5 and 5 years. Wilcoxon signed rank tests were used to evaluate whether the level of combined strategy use was higher at 5 years than at 3.5 years.

#### Relation of Strategy Use and Other Outcome Measures

We hypothesized that local and global ordering strategies are building blocks of number line estimation performance. Higher level strategies should thus be associated with better estimation performance as indicated by more traditional outcome measures.

First, absolute estimation error was used as an indicator of estimation accuracy. The absolute estimation error was calculated for each item by subtracting the target quantity from the estimated quantity and taking the outcome’s absolute value (e.g., when the target quantity was 59 and the child estimated this quantity at position 43 at the line, the absolute estimation error would be 43–59 = 16). Mean absolute estimation error across all items was calculated for each child separately and used as an outcome measure.

Second, model fit of different regression models was considered an indicator of specific estimation patterns. The estimates of each child were regressed onto a linear and a logarithmic model^3^. The linear model is thought to reflect more advanced performance than the logarithmic model (as outlined in the introduction). Thus, we expected higher level local and global ordering strategies to be associated with better fit indices of the linear model. Nevertheless, the logarithmic model was tested as well, because children in the current study might be too young to show linear estimation patterns. The individual model fit index *R*^2^ was used as an outcome measure for both models. We would like to emphasize that we do not want to imply an innate mental number line by testing linear and logarithmic regression models. We used these models only as an index of specific data patterns.

Third, the ordinal relation between the target and estimated quantities was quantified using Kendall’s tau-b. This non-parametric correlation was used as an alternative to the linear and logarithmic model, to simply evaluate the ordering of estimates without imposing a pre-specified model onto the data.

Before analyzing the relation between strategy use and other outcome measures, absolute estimation error, linear fit index, logarithmic fit index, and ordinal relation index were examined separately. Thirty-one (32.0%) 3.5-year-old children and two (4.4%) 5-year-old children showed a negative correlation between target and estimated quantities. Because all negative relations between the target and estimated quantities are considered incorrect estimation patterns, these children were assigned a score of 0 on the linear fit index, logarithmic fit index, and ordinal relation index. Linear fit index, logarithmic fit index, and ordinal relation index were all heavily skewed to the right at 3.5 years^4^. Therefore, Wilcoxon signed ranks tests and Kendall’s tau-b correlation coefficients were used to analyze growth and relation over time of these outcome measures. A dependent samples *t*-test and a Pearson correlation were used to analyze the development of the error score, which was normally distributed at both time points.

The relation between the level of strategy use and the other outcome measures was analyzed using Kendall’s tau-b correlation coefficient.

An α-level of 0.05 was used for all statistical analyses.

## Results

### Strategy Use at 3.5 Years

The frequency distribution of local and global ordering strategy use is depicted in **Table 1**. More than half of the children did not use either a local nor global ordering strategy. For the local ordering strategy, almost all of the remaining children used a local ordering strategy without considering relative distance between quantities (level 1). The variation in level of global ordering strategy use was larger, but the number of children that used each level of the global ordering strategy decreased from level 1 to level 4.

**Table 1:** Frequency of strategy use on the non-symbolic number line at 3.5 years.

	0. No global ordering	1. Global ordering small/large	2. Global ordering small/medium/large	3. Global ordering small quantities	4. Global ordering all quantities	Total
0. No local ordering	52 (53.61)	7 (7.22)	1 (1.03)	0 (0.00)	0 (0.00)	60 (61.86)
1. Local ordering	14 (14.43)	6 (6.19)	6 (6.19)	5 (5.15)	3 (3.09)	34 (35.05)
2. Local ordering relative distance 20%	0 (0.00)	0 (0.00)	1 (1.03)	1 (1.03)	1 (1.03)	3 (3.09)
3. Local ordering relative distance 10%	0 (0.00)	0 (0.00)	0 (0.00)	0 (0.00)	0 (0.00)	0 (0.00)
Total	66 (68.04)	13 (13.40)	8 (8.25)	6 (6.19)	4 (4.12)	97 (100)

**Table 2:** Frequency of strategy use on the non-symbolic number line at 5 years.

	0. No global ordering	1. Global ordering small/large	2. Global ordering small/medium/large	3. Global ordering small quantities	4. Global ordering all quantities	Total
0. No local ordering	6 (13.33)	2 (4.44)	0 (0.00)	0 (0.00)	0 (0.00)	8 (17.78)
1. Local ordering	3 (6.67)	3 (6.67)	7 (15.56)	4 (8.89)	1 (2.22)	18 (40.00)
2. Local ordering relative distance 20%	0 (0.00)	3 (6.67)	6 (13.33)	3 (6.67)	4 (8.89)	16 (35.56)
3. Local ordering relative distance 10%	0 (0.00)	0 (0.00)	0 (0.00)	3 (6.67)	0 (0.00)	3 (6.67)
Total	9 (20.00)	8 (17.78)	13 (28.89)	10 (22.22)	5 (11.11)	45 (100)

**Table 3:** Development of local ordering strategy use on the non-symbolic number line from 3.5 to 5 years.

	5 Years				
3.5 Years	0. No local ordering	1. Local ordering	2. Local ordering relative distance 20%	3. Local ordering relative distance 10%	Total
0. No local ordering	5 (11.63)	9 (20.93)	10 (23.26)	1 (2.33)	25 (58.14)
1. Local ordering	2 (4.65)	7 (16.28)	5 (11.63)	2 (4.65)	16 (37.21)
2. Local ordering relative distance 20%	0 (0.00)	1 (2.33)	1 (2.33)	0 (0.00)	2 (4.65)
3. Local ordering relative distance 10%	0 (0.00)	0 (0.00)	0 (0.00)	0 (0.00)	0 (0.00)
Total	7 (16.28)	17 (39.53)	16 (37.21)	3 (6.98)	43 (100)

**Table 4:** Development of global ordering strategy use on the non-symbolic number line from 3.5 to 5 years.

	5 Years					
3.5 Years	0. No global ordering	1. Global ordering small/large	2. Global ordering small/medium/large	3. Global ordering small numbers	4. Global ordering all numbers	Total
0. No global ordering	6 (13.95)	5 (11.63)	7 (16.28)	4 (9.30)	3 (6.98)	25 (58.14)
1. Global ordering small/large	1 (2.33)	2 (4.65)	1 (2.33)	0 (0.00)	2 (4.65)	6 (13.95)
2. Global ordering small/medium/large	1 (2.33)	0 (0.00)	1 (2.33)	3 (6.98)	0 (0.00)	5 (11.63)
3. Global ordering small numbers	0 (0.00)	0 (0.00)	3 (6.98)	2 (4.65)	0 (0.00)	5 (11.63)
4. Global ordering all numbers	0 (0.00)	1 (2.33)	0 (0.00)	1 (2.33)	0 (0.00)	2 (4.65)
Total	8 (18.60)	8 (18.60)	12 (27.91)	10 (23.26)	5 (11.63)	43 (100)

*Numbers in parentheses indicate percentage of children of the overall sample.*

**Table 5:** Descriptive statistics and pairwise comparisons of absolute estimation error, linear and logarithmic fit indexes and ordinal relation index on the non-symbolic number line at 3.5 and 5 years.

	3.5 Years		5 Years		Pairwise comparison
	M	SD	M	SD	
Absolute estimation error	32.82	8.65	25.08	8.60	*p* < 0.001^c^
Linear fit	0.16 (0.04^a^)	0.21 (0.32^b^)	0.41	0.27	*p* < 0.001^d^
Logarithmic fit	0.18 (0.04^a^)	0.23 (0.39^b^)	0.46	0.27	*p* < 0.001^d^
Ordinal relation	0.21 (0.14^a^)	0.22 (0.36^b^)	0.45	0.23	*p* < 0.001^d^

*N = 97 at 3.5 years, N = 45 at 5 years. ^a^Median. ^b^Interquartile range. ^c^Dependent samples t-test. ^d^Wilcoxon signed rank test.*

**Table 6:** Kendall’s tau-b correlation matrix for the non-symbolic number line at 3.5 and 5 years.

	Local ordering			Global ordering			Strategy combination		
	3.5 Years	5 Years	3.5–5 Years	3.5 Years	5 Years	3.5–5 Years	3.5 Years	5 Years	3.5–5 Years
Absolute estimation error	−0.44^∗∗∗^	−0.47^∗∗∗^	−0.40^∗∗^	−0.42^∗∗∗^	−0.28^∗^	−0.29^∗^	−0.43^∗∗∗^	−0.37^∗∗^	−0.36^∗∗^
Linear fit	0.49^∗∗∗^	0.59^∗∗∗^	0.54^∗∗∗^	0.64^∗∗∗^	0.48^∗∗∗^	0.46^∗∗∗^	0.62^∗∗∗^	0.55^∗∗∗^	0.55^∗∗∗^
Logarithmic fit	0.49^∗∗∗^	0.52^∗∗∗^	0.45^∗∗∗^	0.66^∗∗∗^	0.43^∗∗∗^	0.48^∗∗∗^	0.63^∗∗∗^	0.48^∗∗∗^	0.52^∗∗∗^
Ordinal relation	0.50^∗∗∗^	0.66^∗∗∗^	0.53^∗∗∗^	0.63^∗∗∗^	0.52^∗∗∗^	0.47^∗∗∗^	0.62^∗∗∗^	0.62^∗∗∗^	0.55^∗∗∗^

*N = 97 at 3.5 years, N = 45 at 5 years. ^∗^p < 0.05, ^∗∗^p < 0.01, ^∗∗∗^p < 0.001.*

There was a positive relation between the level of local and global ordering strategy use: τ = 0.54, *p* < 0.001. Most children with a lower level local ordering strategy also used a lower level global ordering strategy. Similarly, children who used a higher level local ordering strategy also used a higher level global ordering strategy. It should be noted, however, that the occurrence of the highest level was quite seldom for global ordering strategies and no child used the highest level of the local ordering strategies.

### Strategy Use at 5 Years

**Table 2** shows the frequency of strategy use on the non-symbolic number line at 5 years of age. For local ordering strategies, almost all children used either local ordering without relative distance (level 1) or local ordering with 20% relative distance strategy (level 2). Again, there was more variation in levels of global ordering strategies. Frequency of strategy use was quite similar across all levels of global ordering strategy use, although there was a slight increase in frequency from level 1 to level 2 and a slight decrease from level 2 to level 4.

Similar to the results at 3.5 years, Kendall’s tau-b showed that levels of local and global ordering strategy use were positively related: τ = 0.53, *p* < 0.001.

### Development in Strategy Use

The development in strategy use was first analyzed for the two strategies separately. In general, higher strategies were used at 5 years than at 3.5 years. Only 13% of the 5-year-old children used none of the strategies, compared to 54% of the 3.5-year-old children. A Wilcoxon signed ranks test showed that there was significant improvement in local ordering strategies from 3.5 to 5 years, *z* = −4.36, *p* < 0.001. Twenty-seven children (63%) used a higher local ordering strategy level at 5 years than at 3.5 years of age, as opposed to 13 children (30%) who used the same strategy level at both time points and three children (7%) who used a lower strategy level at 5 years than at 3.5 years of age (see **Table 3**). Nevertheless, there was no significant relation between children’s strategy use at 3.5 years and their strategy use at 5 years, as indicated by Kendall’s tau-b: τ = 0.07, *p* = 0.606.

The results for global ordering strategy use were similar to the results of local ordering strategy use. Slightly more than half of the children (58%) used a higher global ordering strategy level at 5 years than at 3.5 years of age. Eleven children (26%) used the same strategy at age 3.5 and 5 years of age and seven children (16%) used a lower strategy level at 5 years than at 3.5 years (see **Table 4**). A Wilcoxon signed ranks test showed that the improvement in global ordering strategy level was significant, *z* = −3.45, *p* = 0.001. Again, there was no significant relation between levels of strategy use at 3.5 and 5 years: τ = 0.15, *p* = 0.247.

Next, the interrelation of the development of local and global ordering strategies was investigated by (1) correlating the improvement in local ordering strategy use to the improvement in global ordering strategy use, (2) correlating the combined level of local and global ordering strategy use at 3.5 years with the combined level at 5 years of age, and (3) analyzing the improvement in the combined level of local and global ordering strategy use (see the description of analyses in the Section “Materials and Methods”).

The improvement in local ordering strategies from age 3.5 to 5 years was significantly correlated to improvement in global ordering strategies: τ = 0.49, *p* < 0.001. However, the combined level of strategy use at 3.5 years was not significantly related to the combined level of strategy use at 5 years: τ = 0.12, *p* = 0.325. Nevertheless, a Wilcoxon signed ranks test showed that there was significant improvement in the combined level of strategy use from 3.5 to 5 years: *z* = −3.29, *p* = 0.001. Twenty-nine children (67%) used a higher level combination of strategies at 5 years compared to 3.5 years. Six children (14%) used the same level combination and eight children (19%) used a lower level combination at 5 years than at 3.5 years.

### Relation With Other Outcome Measures

Finally, the relation between strategy use and other (more traditional) outcome measures of the number line estimation task was evaluated. Descriptive statistics and pairwise comparisons for the traditional outcome measures (absolute estimation error, fit indices for linear and logarithmic models, and ordinal relation index) are displayed in **Table 5**. Because linear and logarithmic fit indices as well as the ordinal relation index at 3.5 years were skewed to the right, median and interquartile range are reported for these variables as well. There was significant improvement in performance on all outcome measures (see **Table 5**). Moreover, there was a significant correlation between the ordinal relation index at 3.5 and 5 years, τ = 0.22, *p* = 0.049. Absolute estimation error and linear and logarithmic fit indexes were not significantly correlated over time (*r*_error_ = 0.01, *p* = 0.966, τ_linear_ = 0.16, *p* = 0.147, τ_logarithmic_ = 0.17, *p* = 0.121).

Kendall’s tau-b was used to analyze the relation between strategy use and the other outcome measures. Overall, strategy use at 3.5 and 5 years was significantly related to the other outcome measures (see **Table 6**). Use of higher strategy levels was associated with better performance on the other outcome measures. Moreover, the development in strategy use was positively correlated to the development in the other outcome measures.

## Discussion

In the current study, we extended previous research on the use of local and global ordering strategies in number line estimation by pursuing an in-depth analysis of the development of non-symbolic number line estimation in 3.5- to 5-year-old children. Generally, the results of the current study indicated that about half of the 3.5-year-old children already made use of local and global ordering when estimating non-symbolic quantities on a number line. However, it needs to be considered that the accuracy of their estimations was low with goodness of fit indices of the linear and logarithmic model as well as the ordinal relation index were heavily skewed to the right, with the majority of scores around 0.

This is in line with previous studies on symbolic and non-symbolic number line estimation, revealing that many young children may not yet have developed the underlying skills of number line estimation sufficiently to make valid estimations (Berteletti et al., 2010; Friso-van den Bos et al., 2014a; Praet and Desoete, 2014; Friso-van den Bos et al., 2015). Nevertheless, in the current study, we observed a significant increase in the percentage of children that used local or global ordering strategies from 3.5 to 5 years of age. The percentage of children that used one or both strategies increased from 46% at 3.5 years to 87% at 5 years. Moreover, different levels of local and global ordering strategy use were identified, following a developmental trend from the use of lower level strategies at age 3.5 years to the use of more advanced strategies at age 5 years, which will be discussed in more detail in the following.

### Development of Strategy Use

The developmental trend observed in local ordering in the current study substantiated the results of Sullivan and Barner (2014). At first, young children seem to primarily consider ordinal information to estimate quantities. They seem to decide where to position the target quantity on the number line based on information whether the current quantity is smaller or larger than the preceding quantity. Note that the preceding quantity refers to the quantity that was *presented* directly before the current target quantity and not necessarily the quantity that precedes the current target quantity numerically. Later in development, children then seemed to take into account relative distance between successive quantities. At this stage, they do not only take into account whether the actual target quantity is smaller or larger than the previous item, but also *how much* it is smaller or larger. In the current study, only 3% of the 3.5-year-old children already considered this in their local ordering strategies. Their estimation pattern reflected correct relative distances between successive quantities, within ±20% of the numerical range of the number line. This percentage increased to 36% at age 5 years. Moreover, some (7%) 5-year-olds even made local ordering responses considering correct relative distance between successive quantities within ±10% of the numerical range of the number line. This suggests that there is not only a developmental trend from simple local ordering to local ordering considering relative distance, but also a developmental trend in the degree at which relative distance is considered.

For global ordering, we focused on the ordering of all quantities along the number line, instead of focusing on trial-by-trial ordering. Based on previous research on logarithmic and linear estimation patterns in symbolic number line estimation (e.g., Booth and Siegler, 2006; Friso-van den Bos et al., 2014a), we expected that global ordering should be observed for small quantities first. In other words, early in development children are expected to only order small quantities consecutively, with no or little distinction between larger quantities. This will be followed by global ordering of the whole range of quantities later in development.

The data partially substantiated our expectation of a developmental trend from global ordering of small quantities to global ordering of all quantities. Both levels of global ordering (ordering small quantities and ordering all quantities) were indeed observed, but a clear developmental trend from ordering small quantities to ordering all quantities was not observed. Generally, frequency of these levels of global ordering was low, especially at 3.5 years of age. It turned out that most 3.5-year-old children only distinguished between small and large quantities or between small, medium, and large quantities in global ordering. At age 5 years, more children were able to differentiate between small quantities or even ordered the whole range of quantities consecutively, but it is likely that the broader developmental transition from ordering small quantities to ordering all quantities takes place beyond the age of the children assessed in the current study.

Despite the clear improvement in estimation performance in non-symbolic number line estimation from 3.5 to 5 years, neither local or global ordering strategies nor the more traditional outcome measures at 3.5 years were significantly associated with scores at 5 years. This might suggest that the non-symbolic number line task may not measure the same skills at 3.5 and 5 years of age. An alternative explanation for the observed low correlations may be that the way children solve non-symbolic number line estimation *changes* over time. All children showed improvement in their number line estimation performance, but their improvement as well as their future performance could not be predicted significantly by their estimation accuracy at 3.5 years. In this context, it is important to note that many of the 3.5-year-old children did not use local or global ordering estimation strategies at all whereas they did at 5 years—but at various levels. Nevertheless, the correlation between the ordinal relation index at 3.5 and 5 years was significant. This seems to indicate that there is some continuity in the degree of ordering quantities along the number line from age 3.5 to 5 years.

### The Relation Between Local and Global Ordering Strategies

Importantly, the present results indicated that local and global ordering strategies do not develop in isolation from each other. We observed that levels of local and global ordering strategy use were highly correlated. This means that with increasing level of local ordering strategy children also used a higher level of global ordering and vice versa. This could indicate that *together* local and global ordering act as building blocks of number line estimation performance. However, it is not yet clear whether the association between local and global ordering is caused by developmental processes or is a necessary artifact of the operationalization of the two strategies. It might be possible that global ordering is not possible without local ordering. Nevertheless, the data seems to indicate that local and global ordering do not necessarily need to reflect the same level of proficiency at each time point. Some children used no local ordering but did use global ordering or vice versa. Some children even used one of the lower levels of one strategy and one of the higher levels of the other strategy.

To clarify the issue of dependency of the strategies, we ran some simulations (see **Appendix 1** for the simulation procedure and results). In particular, we simulated local ordering at different levels (i.e., 100 simulated participants for each level of local ordering) and then coded global ordering for these simulated estimation patterns. The results of this simulation were similar to the results observed in our data. The correlation (as indicated by Kendall’s tau-b) between simulated local and global ordering strategies was 0.68. Despite this high correlation, the frequency table of simulated strategies showed considerable variation in the level of global ordering strategies within each level of local ordering, except for local ordering level 0. For local ordering level 0, only 6 out of 100 simulated estimation patterns were coded as global ordering. This might lead to the conclusion that various levels of global ordering arise from local ordering by chance. However, both the simulation data and the participants’ data also showed that it is difficult, but not impossible, to achieve global ordering without local ordering. This seems to substantiate that, although local and global ordering are related, they are not just two sides of the same coin.

Furthermore, an important theoretical distinction between the two strategies can be made. Local ordering assumes the use of previous estimates as reference points, whereas global ordering assumes the use of external reference points, like the beginning or endpoint of the number line. For example, in global ordering level 1, small and large quantities are distinguished, with large quantities positioned rightward of small quantities. This requires relating quantities to the beginning and/or endpoint of the line to decide where to position small and large quantities on the number line.

Nevertheless, it is possible that local and global ordering strategies become more integrated over time. The results of the current study showed that children’s local ordering strategies developed from ordering successive quantities toward taking into account the relative distance between quantities. Estimating relative distance requires taking into account the length of the number line to estimate the proportion of the line that corresponds to the relative distance between quantities. Therefore, quantities have to be related to external reference points on the number line, like the beginning and endpoint of the line. When the distance between these external reference points is not taken into account it would probably not be possible to estimate the correct proportion of the number line that corresponds to the relative distance between quantities. This resembles first steps toward proportion-based estimation strategies, which have previously been demonstrated to be solution strategies in number line estimation, as indicated by fitting of cyclic power models to estimation patterns (e.g., Barth and Paladino, 2011). The current study extends these previous findings by suggesting that proportion-based estimation strategies may also incorporate previous estimates as reference points.

In the current study, cyclic power models could not be identified reliably. These models probably require more advanced proportional reasoning. So far, reliable fit of cyclic power models was usually found from first grade onward (e.g., Friso-van den Bos et al., 2015). We hypothesize that children will increasingly make use of both previous estimates and external benchmarks on the number line as reference points for estimation throughout development. Together, local and global ordering strategies should act as building blocks of number line estimation. In line with this notion, the current study indicated that higher level local and global ordering was associated with improved estimation performance in non-symbolic number line estimation. Children who used higher levels of local and global ordering also showed higher estimation accuracy, higher logarithmic and linear fit scores, as well as a higher ordinal relation between target and estimated quantities on their non-symbolic number line estimation at 3.5 and 5 years of age. Future research is needed to further investigate the interplay between using previous estimates and external reference points, in order to better understand the relation between local and global ordering, and their role as building blocks of number line estimation performance, throughout development.

Nevertheless, our findings support the view that estimates may be formed during task execution (Cohen and Sarnecka, 2014; Cohen and Quinlan, 2018), and seem to offer an alternative explanation of seemingly logarithmic and linear estimation patterns found in the current study. For example, global ordering of small quantities without differentiating between larger quantities (i.e., global ordering level 3 in the current study) would result in a seemingly logarithmic estimation pattern. Similarly, global ordering of all quantities would result in a seemingly linear estimation pattern. Therefore, even though strategy use was related to logarithmic and linear fit scores in the current study, these estimation patterns may not necessarily reflect mental number line representations, but can instead be explained by strategy use. The current study thus showed that number line estimation does not seem to be a unidimensional construct, but rather builds on interacting strategies, stressing the importance of on-task processing and strategy use instead of mental number line representations.

### Underlying Mechanisms of Strategy Use

The important role of dynamic ordering strategies in number line estimation might suggest that children’s estimates are primarily guided by ordinal processes at these early ages. Nevertheless, other processes might play a role in local and global ordering strategy use as well. Although the underlying mechanisms of strategy use were not investigated in the current study, we would like to make some suggestions based on previous research to specify potential starting points for further research.

As discussed above, refining estimation of relative position of quantities on a number line probably requires general cognitive skills like analogical and proportional reasoning as well (e.g., Barth and Paladino, 2011; Sullivan and Barner, 2014). Moreover, the use of reference points probably also requires working memory, as for example participants have to remember the position of previous estimates. Therefore, we hypothesize that general cognitive skills play an important role in number line estimation.

Nevertheless, domain-specific numerical skills are also needed to estimate numbers on a number line. It is likely that both ordinality and cardinality are underlying mechanisms in children’s non-symbolic number line estimation. To use local ordering, mainly ordinal information is used as participants compare the target quantity to the preceding quantity and decide which one is smaller and which one is larger. For example, when ordering quantities 71 and 75, participants have to understand that the second quantity is larger than the first, but not necessarily that the first quantity is 71 and the second quantity is 75. For global ordering, cardinal processes might play an important role. Results of the current study showed that in general smaller quantities were placed closer to the beginning point and larger quantities were placed closer to the endpoint of the number line. This was already observed at the lowest levels of global ordering. This might indicate that in global ordering children considered not only the relation between quantities, but also the actual magnitudes when considering the relative distance between quantities and external reference points of the number line. This is in line with propositions in previous research on the use of proportional reasoning strategies in number line estimation (e.g., Barth and Paladino, 2011; Sullivan and Barner, 2014). Even if participants did not estimate the correct cardinal position on the line, this suggests that the cardinal value of each quantity is considered when estimating relative distance between the target quantity and external reference points on the number line. Interestingly, Lyons and Beilock (2013) proposed that non-symbolic ordinal tasks are actually solved through considering cardinality as well. As such, in local ordering non-symbolic quantities may be ordered by comparing the cardinal value of each quantity with the preceding quantity, instead of relating the quantities to their “neighbors.” More research is needed to clarify the role of cardinality and ordinality in non-symbolic number line estimation.

Another skill that is probably needed for number line estimation is visual discrimination of quantities and classification of the difference between quantities in terms of smaller and larger. In case a child cannot discriminate between quantities, she/he would not be able to place quantities on the line in an ordered manner. However, the relation between quantity discrimination and non-symbolic number line estimation is not yet clear. Some studies have shown that non-symbolic quantity discrimination and non-symbolic number line estimation are associated (Kolkman et al., 2013; Friso-van den Bos et al., 2014b), while others have proposed that these tasks rely on different underlying mechanisms (Sasanguie and Reynvoet, 2013). Further research is needed to evaluate the relation between quantity discrimination and non-symbolic number line estimation.

### Limitations of the Current Study

When interpreting the results of the current study it is important to note that almost all participants were from rather high SES families. This limits the external validity of the current study to other SES classes, because cognitive performance was found to be influenced by SES (e.g., Jordan et al., 2006). Furthermore, it is not known to what extent children in the current study were able to discriminate between respective quantities. As mentioned above, discrimination of quantities might be related to number line estimation performance. Previous research showed that 3-year-olds are able to discriminate quantities at the ratio of 3:4 and 5-year-olds are able to discriminate quantities at the ratio of 4:5 (Halberda and Feigenson, 2008). However, the results of Halberda and Feigenson (2008) might not be transferable to our items easily, because Halberda and Feigenson (2008) controlled their stimuli for non-numerical cues like surface area, etc., whereas stimuli in the current study were not controlled for these cues. Instead, dot size was kept constant across stimuli, which resulted in a positive association between numerical quantity and total surface area (i.e., larger quantities cover a larger total surface area). In previous research, visual-spatial cues associated with numerical quantity were controlled in non-symbolic stimuli to make children attend to numerical quantity instead of continuous extent (introduced by Clearfield and Mix, 1999). However, the ecological validity of such controlled stimuli might be low. Instead, it is likely that visual-spatial extent and numerical quantity are hard to separate (see Leibovich et al., 2017, for a discussion). Following Cantrell and Smith (2013), we argue that the association between continuous extent and numerical quantity may not be a problem, but makes numerical quantity more salient to participants on non-symbolic numerical tasks, as an ecologically valid aid. As such, children should have had fewer difficulties discriminating quantities in the current study.

## Conclusion

Taken together, the current study provides a new perspective on number line estimation in early childhood. The results indicate that the seemingly logarithmic (and linear) patterns found in previous research do not necessarily represent static mental number representations, but may instead be explained by children’s dynamic ordering strategies while performing the task. Furthermore, it suggests that the logarithmic estimation pattern often observed for young children and unfamiliar number ranges (e.g., Siegler and Opfer, 2003; Siegler and Booth, 2004) does not seem to be the most basic form of number line estimation. Even before children can order small quantities consecutively, they are able to differentiate between small and large or small, medium and large quantities on the number line. Non-symbolic number line estimation hence builds on the use of local and global ordering strategies, which are already present at 3.5 years of age. These strategies develop from simply considering the ordinality of target quantities to more complex levels of local and global ordering strategies also considering first aspects of cardinality and proportional reasoning between the age of 3.5 and 5 years.

Importantly, we suggest that these strategies represent building blocks, not an end stage of non-symbolic number line estimation. Local and global ordering strategies as measured in the current study may only represent early and basic levels of strategy use. For example, the highest level of global ordering in the current study was assigned when a child ordered all quantities correctly, even when relative distance between quantities or the cardinal position of quantities on the number line was not correct. Considering these aspects would require further development of the respective strategy levels. Future research may therefore aim to incorporate correct ordering as well as correct relative distance between quantities and correct (cardinal) position on the number line, in particular when studying older children. Furthermore, as symbolic number skills become more important during primary school, it would be desirable to also investigate the generalizability of local and global ordering strategies to symbolic number line estimation.

## Author Contributions

JVN, MV, PL, and EK contributed to the outline of this research. JVN collected the data and supervised the study. All authors contributed to data analysis and writing.

## Conflict of Interest Statement

The authors declare that the research was conducted in the absence of any commercial or financial relationships that could be construed as a potential conflict of interest.

## Footnotes

1 A first draft of this article was published as part of the first author’s doctoral thesis (van ’t Noordende, 2018).

2 We choose to use Kendall’s tau-b in all analyses considering non-parametric correlations, because it is usually preferred over Spearman’s non-parametric correlation for small data sets and data with a large number of tied ranks (cf. Field, 2013).

3 Power models were also tested (cf. Barth and Paladino, 2011; Rouder and Geary, 2014), but one-cycle and two-cycle power models could not be identified for most children. The results of non-cyclic power model were largely identical to those of the logarithmic model. Therefore, power models were not considered here.

4 The skewed distribution of the linear and logarithmic *R*^2^ was not caused by the scores of 0 that were assigned to children with a negative relation between the target and estimated quantities. The distribution did not change significantly when using the original scores or when excluding these scores. The distribution of Kendall’s tau-b was altered by assigning the scores of 0, but the results of the analyses did not change.

## Appendix 1

The computer program R was used to simulate each level of local ordering, using the following codes, in which the variable *x* refers to the target quantities and the variable *y* refers to simulated estimates:

Local ordering level 0:

x < - sample(c(6, 14, 21, 27, 33, 39, 47, 52, 59, 71, 76, 84, 90, 95), 14)

y < - sample(c(0:100), 14)

Local ordering level 1:

x < - sample(c(6, 14, 21, 27, 33, 39, 47, 52, 59, 71, 76, 84, 90, 95), 14)

y1 < - sample(0:100, 1)

if (x[2] < x[1]) y2 < - sample(c((0:(y1-(abs(x[2]-x[1]) + 20))), ((y1-(abs(x[2]-x[1]) - 20)):(y1-5))), 1)

if (x[2] > x[1]) y2 < - sample(c((y1+5):(y1+(abs(x[2]-x[1]) - 20)), ((y1+(abs(x[2]-x[1]) + 20)):100)), 1)

if (y2 < 0) y2 < - 0

if (y2 > 100) y2 < - 100

if (x[3] < x[2]) y3 < - sample(c((0:(y2-(abs(x[3]-x[2]) + 20))), ((y2-(abs(x[3]-x[2]) - 20)):(y2-5))), 1)

if (x[3] > x[2]) y3 < - sample(c((y2+5):(y2+(abs(x[3]-x[2]) - 20)), ((y2+(abs(x[3]-x[2]) + 20)):100)), 1)

if (y3 < 0) y3 < - 0

if (y3 > 100) y3 < - 100

And so on for all 14 items.

Local ordering level 2:

x < - sample(c(6, 14, 21, 27, 33, 39, 47, 52, 59, 71, 76, 84, 90, 95), 14)

y1 < - sample(0:100, 1)

if (x[2] < x[1]) y2 < - y1 - sample((abs(x[2]-x[1]) - 20):(abs(x[2]-x[1]) + 20), 1)

if (x[2] > x[1]) y2 < - y1 + sample((abs(x[2]-x[1]) - 20):(abs(x[2]-x[1]) + 20), 1)

if (y2 < 0) y2 < - 0

if (y2 > 100) y2 < - 100

if (x[3] < x[2]) y3 < - y2 - sample((abs(x[3]-x[2]) - 20):(abs(x[3]-x[2]) + 20), 1)

if (x[3] > x[2]) y3 < - y2 + sample((abs(x[3]-x[2]) - 20):(abs(x[3]-x[2]) + 20), 1)

if (y3 < 0) y3 < - 0

if (y3 > 100) y3 < - 100

And so on for all 14 items.

Local ordering level 3:

x < - sample(c(6, 14, 21, 27, 33, 39, 47, 52, 59, 71, 76, 84, 90, 95), 14)

y1 < - sample(0:100, 1)

if (x[2] < x[1]) y2 < - y1 - sample((abs(x[2]-x[1]) - 10):(abs(x[2]-x[1]) + 10), 1)

if (x[2] > x[1]) y2 < - y1 + sample((abs(x[2]-x[1]) - 10):(abs(x[2]-x[1]) + 10), 1)

if (y2 < 0) y2 < - 0

if (y2 > 100) y2 < - 100

if (x[3] < x[2]) y3 < - y2 - sample((abs(x[3]-x[2]) - 10):(abs(x[3]-x[2]) + 10), 1)

if (x[3] > x[2]) y3 < - y2 + sample((abs(x[3]-x[2]) - 10):(abs(x[3]-x[2]) + 10), 1)

if (y3 < 0) y3 < - 0

if (y3 > 100) y3 < - 100

And so on for all 14 items.

**Table 7:** This resulted in the following frequency table:

	0. No global ordering	1. Global ordering small/large	2. Global ordering small/medium/large	3. Global ordering small quantities	4. Global ordering all quantities	Total
0. No local ordering	94 (23.50)	6 (1.50)	0 (0.00)	0 (0.00)	0 (0.00)	100 (25.00)
1. Local ordering	30 (7.50)	42 (10.50)	26 (6.50)	2 (0.50)	0 (0.00)	100 (25.00)
2. Local ordering relative distance 20%	3 (0.75)	16 (4.00)	59 (14.75)	16 (4.00)	6 (1.50)	100 (25.00)
3. Local ordering relative distance 10%	1 (0.25)	13 (3.25)	56 (14.00)	11 (2.75)	19 (4.75)	100 (25.00)
Total	33 (8.25)	80 (20.00)	130 (32.50)	29 (7.25)	28 (7.00)	400 (100)

*Numbers in parentheses indicate percentage of children of the overall sample.*
